# High-resolution synchrotron X-ray study of icosahedrite, an icosahedral AlCuFe quasicrystal from the Khatyrka meteorite

**DOI:** 10.1107/S2052252525004130

**Published:** 2025-05-23

**Authors:** H. Takakura, K. Mizunuma, T. Yamada, A. Bosak, F. Formisano, L. Paolasini, M. de Boissieu, P. J. Steinhardt, L. Bindi

**Affiliations:** a Faculty of Engineering, Division of Applied Physics Hokkaido University Hokkaido Japan; b Graduate School of Engineering, Division of Applied Physics Hokkaido University Hokkaido Japan; c Faculty of Advanced Engineering, Department of Applied Physics Tokyo University of Science Tokyo125-8585 Japan; d European Synchrotron Radiation Facility Grenoble France; e CNR-IOM & INSIDE@ILL c/o Operative Group in Grenoble (OGG) and Institut Laue Langevin (ILL) GrenobleF-38042 France; f Université Grenoble Alpes, CNRS, Grenoble INP, SIMaP GrenobleFrance; g Department of Physics Princeton University Princeton NJ08544 USA; h Department of Earth Sciences University of Florence FlorenceI-50121 Italy; IRCP Chimie-ParisTech, France

**Keywords:** icosahedrite, quasicrystals, icosahedral modulated phases, phasons, Khatyrka meteorite

## Abstract

Icosahedrite, natural icosahedral Al_63_Cu_24_Fe_13_, was discovered in a meteorite about 15 years ago. We have carried out a high-resolution X-ray diffraction study on a sample of this meteoritic mineral at the ESRF. The diffraction pattern turned out to be identical to an intermediate phase observed in synthetic *i*-AlCuFe during the transformation from the quasicrystalline state to a periodic rhombohedral phase. This particular natural Al_63_Cu_24_Fe_13_ grain is an icosahedral quasicrystal on which a modulation by six cosine waves propagating along the fivefold axes is superimposed, with a wavelength of about 20 nm and a polarization in the phason/perpendicular space. By examining the thermodynamic conditions for producing this modulated icosahedral phase at high pressure in the laboratory, we may gain insights into the formation process of the Khatyrka meteorite.

## Abstract 2

A single grain of the icosahedral AlCuFe quasicrystal extracted from the Khatyrka meteorite has been studied by means of high-resolution synchrotron X-ray diffraction at the ESRF. We found that the mineral is a phason-wave modulated icosahedral quasicrystal, a feature already observed for synthetic quasicrystals. This result might be used as a tracer to shed light on the thermal history of the meteorite.

## 1. Introduction

Aperiodic crystals are long-range-ordered materials that lack lattice periodicity in at least one direction [see Janssen *et al.* (2018 ▸) for an introduction on aperiodic crystals]. They are characterized by a diffraction pattern with sharp Bragg reflections and require more than three integer indices for proper indexing. This is the basis of the superspace description of aperiodic crystals, where periodicity is recovered in a space with more than three dimensions (Janssen & Janner, 2014 ▸). Aperiodic crystals are generally classified into three categories (Janssen *et al.*, 2018 ▸): incommensurately modulated phases; incommensurate composite phases; and quasicrystals, discovered most recently (Shechtman *et al.*, 1984 ▸; Levine & Steinhardt, 1984 ▸). Aperiodic crystals are found almost everywhere in hard and soft condensed matter: simple elements under pressure, minerals, intermetallics, oxides, organic compounds, micelle solutions, polymers and even protein crystals (Janssen *et al.*, 2018 ▸). Aperiodic crystals are also found in many minerals (Bindi *et al.*, 2020 ▸). Calaverite is the first mineral that attracted much attention because the indexing of its crystal faces was violating Haüy’s laws. It turns out that its structure is an incommensurately modulated phase [see Janssen & Janner (2014 ▸) and references therein]. Incommensurately modulated phases are in fact regularly found in minerals (Bindi *et al.*, 2020 ▸), and re-investigation of well known minerals such as labradorite feldspar (Jin *et al.*, 2020 ▸) can lead to a solution in terms of a modulated structure. They are also observed for minerals under pressure (Gajda *et al.*, 2025 ▸). A few incommensurate composites have also been observed (Evain *et al.*, 2006 ▸).

About 15 years ago icosahedrite – the first natural quasicrystal – was discovered in a meteorite: it has the composition Al_63_Cu_24_Fe_13_ with an icosahedral symmetry (Bindi *et al.*, 2009 ▸, 2011 ▸). Its electron diffraction pattern displays all the characteristics of an icosahedral quasicrystal, being almost identical to the synthetic *i*-AlCuFe quasicrystal discovered by Tsai *et al.* (1987 ▸). Detailed studies of the meteorite containing icosahedrite and shock experiments have demonstrated that icosahedrite formed by an impact-induced shock in outer space, where temperatures larger than 1400 K and pressures around 5–10 GPa were reached (Bindi *et al.*, 2012 ▸; Hollister *et al.*, 2014 ▸; Asimow *et al.*, 2016 ▸; Lin *et al.*, 2017 ▸; Meier *et al.*, 2018 ▸; Tommasini *et al.*, 2021 ▸). However, in a collision between asteroids, the impact generates highly varying pressure and temperature conditions in the collided material due to the extreme and localized nature of the event. The force of the collision causes rapid compression and heating in some areas, while others experience lower pressures and temperatures. These variations can result in significant heterogeneities, with some regions undergoing intense shock melting and others remaining relatively unaffected. Such disparities play a critical role in shaping the physical and chemical characteristics of the resulting materials, as well evident in the Khatyrka meteorite (Lin *et al.*, 2017 ▸; Meier *et al.*, 2018 ▸; Tommasini *et al.*, 2021 ▸).

The synthetic *i*-AlCuFe quasicrystal was one of the first so-called stable quasicrystals. It can be obtained by slow cooling from the melt and results in millimetre-size single-grain crystals (Tsai *et al.*, 1987 ▸). The Al–Cu–Fe phase diagram is complex and the quasicrystal forms via a peritectic growth (Bradley & Goldscmidt, 1939 ▸; Dong *et al.*, 1990 ▸; Faudot *et al.*, 1991 ▸; Gayle *et al.*, 1992 ▸; Dong *et al.*, 1989 ▸). Single grains obtained by slow cooling exhibit the Al_63.5_Cu_24_Fe_12.5_ composition and are in the icosahedral quasicrystalline state only at temperature above 675°C. When slowly cooled from the high-temperature phase, the quasicrystal transforms into a large periodic rhombohedral approximant with the lattice parameter *a*_R_ = 3.216 nm and a rhombohedral angle equal to 36° (Audier & Guyot, 1989 ▸, 1990 ▸; Audier, 1990 ▸). The low-temperature phase is actually a multi-domain structure with domains of about 20 nm in size, which are coherently oriented along each of the ten threefold axes of the icosahedron as shown by Audier & Guyot (1989 ▸, 1990 ▸) and Audier (1990 ▸). The large rhombohedral unit cell and the small size of each domain make it difficult to precisely characterize this micro-crystalline state. Moreover, the phase transition is accomplished via a series of intermediate states among which is a modulated icosahedral phase (Audier *et al.*, 1991 ▸) characterized by satellite reflections that lie along the twelve fivefold directions around each of the icosahedral Bragg peak of the high-temperature phase. An X-ray synchrotron radiation diffraction analysis has demonstrated that this modulation can be interpreted as a simple sine wave modulation propagating along the fivefold axis with a wavelength of 19 nm, and with a polarization in the perpendicular phason space (Menguy *et al.*, 1993 ▸*b*, ▸*a*). This transition is reversible, as shown by *in situ*X-ray diffraction (Bancel, 1989 ▸, 1991 ▸, 1993 ▸; Boudard *et al.*, 2000 ▸). It is consistent with a phason-driven phase transition with a characteristic shape of the diffuse scattering due to phason fluctuations that soften when approaching the phase transition (Boudard *et al.*, 2000 ▸), leading to the modulated phase. The latter is then followed by a pentagonal state that finally transforms into the rhombohedral periodic approximant (Menguy *et al.*, 1993 ▸*c*).

To clarify the structural characteristics of the first natural quasicrystal, we have undertaken a high-resolution synchrotron X-ray diffraction study of a small single grain of icosahedrite on the ID28 beamline at the ESRF. The high resolution is mandatory in order to look for possible modulations or microcrystalline states, whereas the high flux is important to measure precisely any diffuse scattering that might originate from phason fluctuations typically observed in all icosahedral synthetic quasicrystals (de Boissieu *et al.*, 1995 ▸; Létoublon *et al.*, 2001 ▸; de Boissieu, 2012 ▸; Yamada *et al.*, 2016 ▸).

## 2. Experimental methods

A single grain of icosahedrite has been selected from the holotype material belonging to the Natural History Museum of the University of Florence, Italy (sample No. 46407/G) and mounted on a glass capillary. The single grain has a prismatic shape with an average dimension equal to 40 × 40 × 60 µm. The X-ray diffraction experiment has been carried out on the side station of the ID28 beamline at ESRF, with a four-circle Eulerian diffractometer and a hybrid pixel Pilatus3 detector in a setup optimized to minimize all parasitic background in order to measure weak Bragg peaks and diffuse scattering (Girard *et al.*, 2019 ▸). The incident X-ray beam is monochromated by a (311) diamond Laue crystal followed by a (422) silicon Bragg crystal. The incident X-ray beam wavelength was set to 0.05701 nm and the beam focused with the combination of a transfocator (Be lenses) and a gradient multilayer mirror with a beam size at the sample position of about ∅30 µm. The threshold of the detector was set up in such a way to supress all the Cu and Fe fluorescences.

Data collection has been carried out with the shutterless continuous phi rotation over 360° mode, with an image acquisition every 0.1°. Two detector angles and two sample-to-detector distances (244 or 414 mm) have been used. For each detector position, measurements with and without attenuation have been carried out to evaluate the very strong and weak Bragg peaks correctly. The constant phi rotation speed was equal to 2.5 and 5 s per degree for measurements without and with attenuation, respectively.

Using a 50 µm collimator, various parts along the sample *z* direction have been explored. All the data displayed a similar diffraction pattern containing reflections of a main quasicrystalline icosahedrite grain, a smaller one and a crystalline grain. We found that a beam position located at the tip of the sample gave a maximum signal for the main icosahedrite grain. In the following, all data presented correspond to this tip sample position.

With this setup, the beam divergence is of the order of 0.01°. The instrumental resolution in the transverse direction depends on the wavevector modulus *Q*, and for a value of *Q* = 3 Å^−1^, the overall resolution is on the order of 0.005 Å^−1^ in all three directions.

## 3. Results

### 3.1. Indexing of the diffraction pattern

Indexing of quasicrystal diffraction patterns can be carried out using the X-ray diffraction data measurement and processing software *CrysAlisPro*. However, the instrumental parameters have to be determined first using *CrysAlisPro* from a zeolite crystal measured at the same conditions. Using this procedure, we determined all the instrumental parameters and then fixed them for further measurements. Indexing of the diffraction pattern of icosahedrite was done with a rhombohedral cell with the lattice parameter *a*_R_ = 0.246 nm and a rhombohedral angle equal to 108°. Additionally, three modulation vectors, which are described as (τ^−1^, −1, τ^−1^), (1, τ^−1^, −τ^−1^) and (τ^−1^, τ^−1^, −1) in the rhombohedral reciprocal lattice were selected. In this way, six basic vectors necessary for indexing the icosahedral quasicrystal were obtained. The icosahedral space group is found to be *Fm*35. From the rhombohedral lattice parameter and after a proper τ^3^ scaling we found that the 6D lattice parameter is equal to *a*_6D_ = 2 × 0.628 nm. Indexing of the reflections is done based on this lattice parameter throughout this paper.

X-ray Bragg peaks were indexed following the scheme proposed by Elser (1986 ▸) and Cahn *et al.* (1986 ▸). For high-symmetry Bragg peaks the short N/M notation (Cahn *et al.*, 1986 ▸) is used instead of the required six integer indices. High-quality reciprocal space sections were prepared with locally developed software using the *CrysAlis*-produced corrections list.

**Figure 1:**
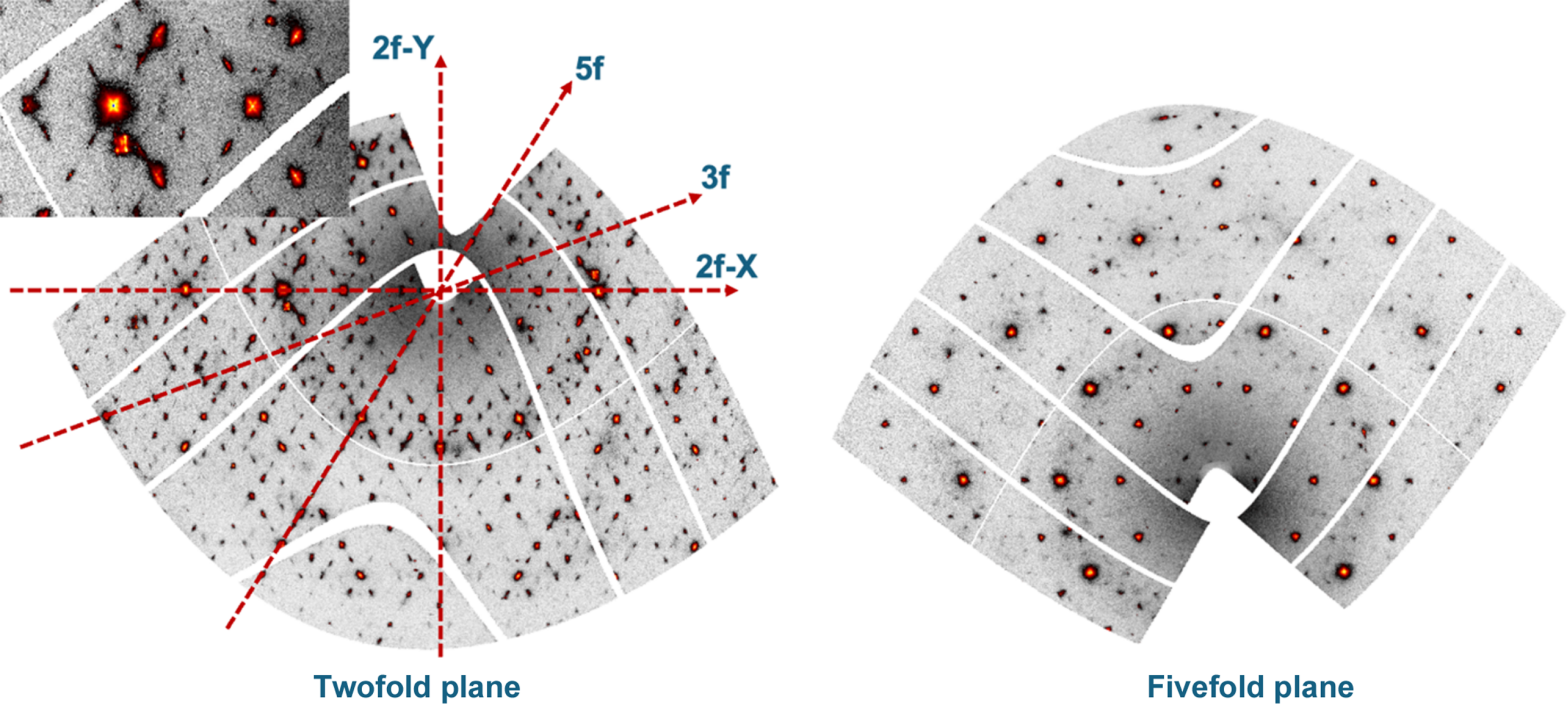
Reconstructed twofold and fivefold diffraction planes of the icosahedrite after **UB** matrix determination and using locally developed software. The sample-to-detector distance is equal to 244 mm. The main two-, three- and fivefold directions are indicated with red dashed lines. On the twofold reciprocal space plane, streaks parallel to the two fivefold axes are clearly visible. The inset shows an enlarged part of the twofold plane. The colour coding and the logarithmic scale are chosen to highlight the weak reflections.

**Figure 2:**
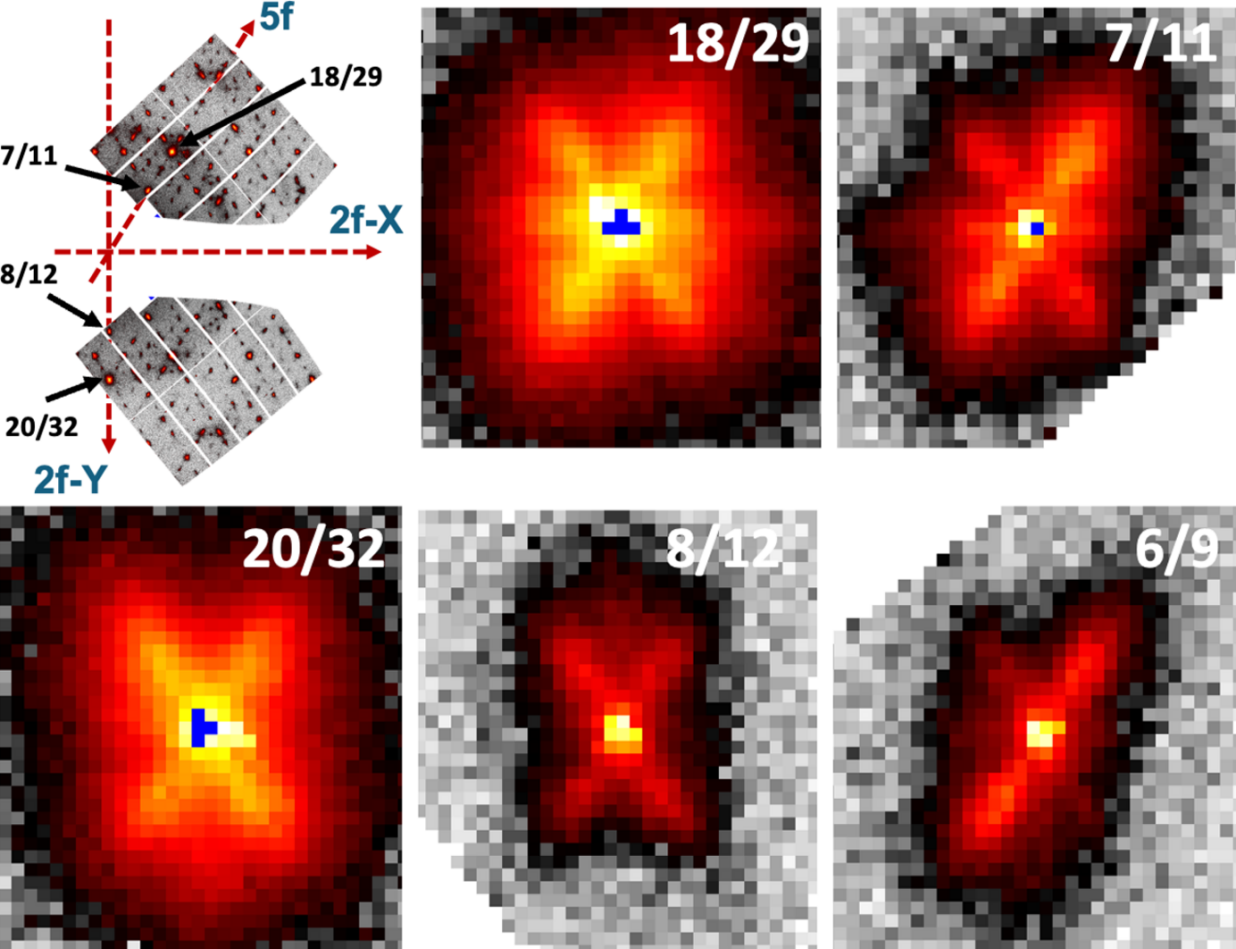
Zoom around a few reflections in the twofold diffraction plane, taken with a sample-to-detector distance equal to 414 mm, allowing a higher *Q*-resolution without attenuation. The first panel (top left) displays the reciprocal space spanned in this configuration, together with the two- and fivefold axes orientations. Then, from top left to bottom right are Bragg peaks that lie on a fivefold axis (18/29 and 7/11), a twofold axis (20/32 and 8/12) and a threefold axis (6/9). All panels are the same size: 1.66 nm^−1^ wide and 1.84 nm^−1^ high. The colour coding and the logarithmic scale are chosen to highlight the weak reflections: first a grey scale for very weak counts, followed by a temperature scale. Pixels with intensity larger than 10^6^ counts are indicated in blue.

**Figure 3:**
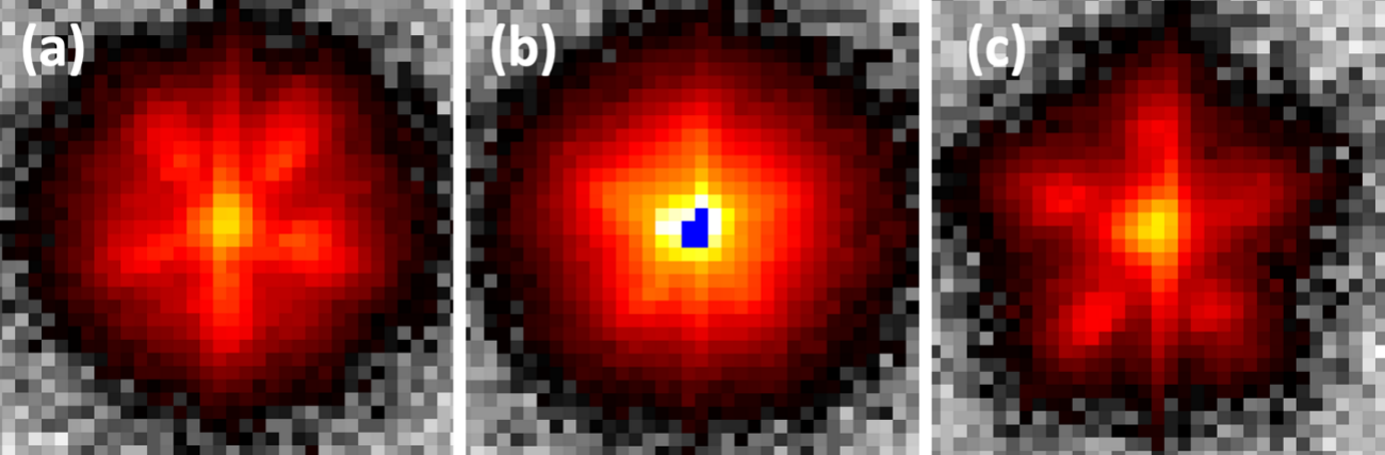
Slices taken around the fivefold 18/29 main Bragg peak along different heights along a fivefold axis: (*a*) −Δ, (*b*) 0 and (*c*) +Δ (Δ = 0.358 nm^−1^). The characteristic pentagon and its mirror image are observed as a signature of the icosahedron shape. All panels are squares with sides of 1.84 nm^−1^. The sample-to-detector distance, size of each panel and colour coding are the same as in Fig. 2 ▸.

Fig. 1 ▸ shows two reciprocal space sections obtained after the determination of the **UB** matrix, the short sample-to-detector distance and using the aforementioned locally developed software. On the left side the twofold diffraction pattern is displayed, with the twofold, threefold and fivefold axes indicated. The right panel displays the fivefold diffraction planes with the characteristic tenfold symmetry. When looking closer at the twofold diffraction pattern (inset panel in Fig. 1 ▸), supplementary satellite reflections around each Bragg peak along directions parallel to the fivefold axis can be observed. This is best exemplified in Fig. 2 ▸, which displays an enlarged view of the reciprocal space around a series of main Bragg peaks. The figures have been realized using the largest detector-to-sample distance, which allows a better *Q*-resolution. All reflections are displayed with the same axis orientations in a twofold plane as shown in the first panel (left). Bragg peaks are labelled with their N/M indices and the area displayed covers a rectangle of 1.66 nm^−1^ by 1.84 nm^−1^. The Bragg peaks lie along a fivefold axis (18/29 and 7/11), a twofold axis (20/32 and 8/12) and a threefold axis (6/9). For all Bragg peaks there are supplementary satellite reflections that lie along directions parallel to the fivefold axis. If satellites lie along the fivefold axis, we should thus observe 12 satellites that lie on the vertices of an icosahedron around each Bragg peak. This is indeed shown by observing successive reciprocal layers along a direction perpendicular to a fivefold axis as illustrated in Fig. 3 ▸. The position with respect to the central part is indicated by Δ. One clearly observes a pentagon and its mirror image at +Δ and −Δ, as expected for an icosahedron. Each Bragg peak is thus surrounded by twelve satellites in an icosahedral arrangement.

**Figure 4:**
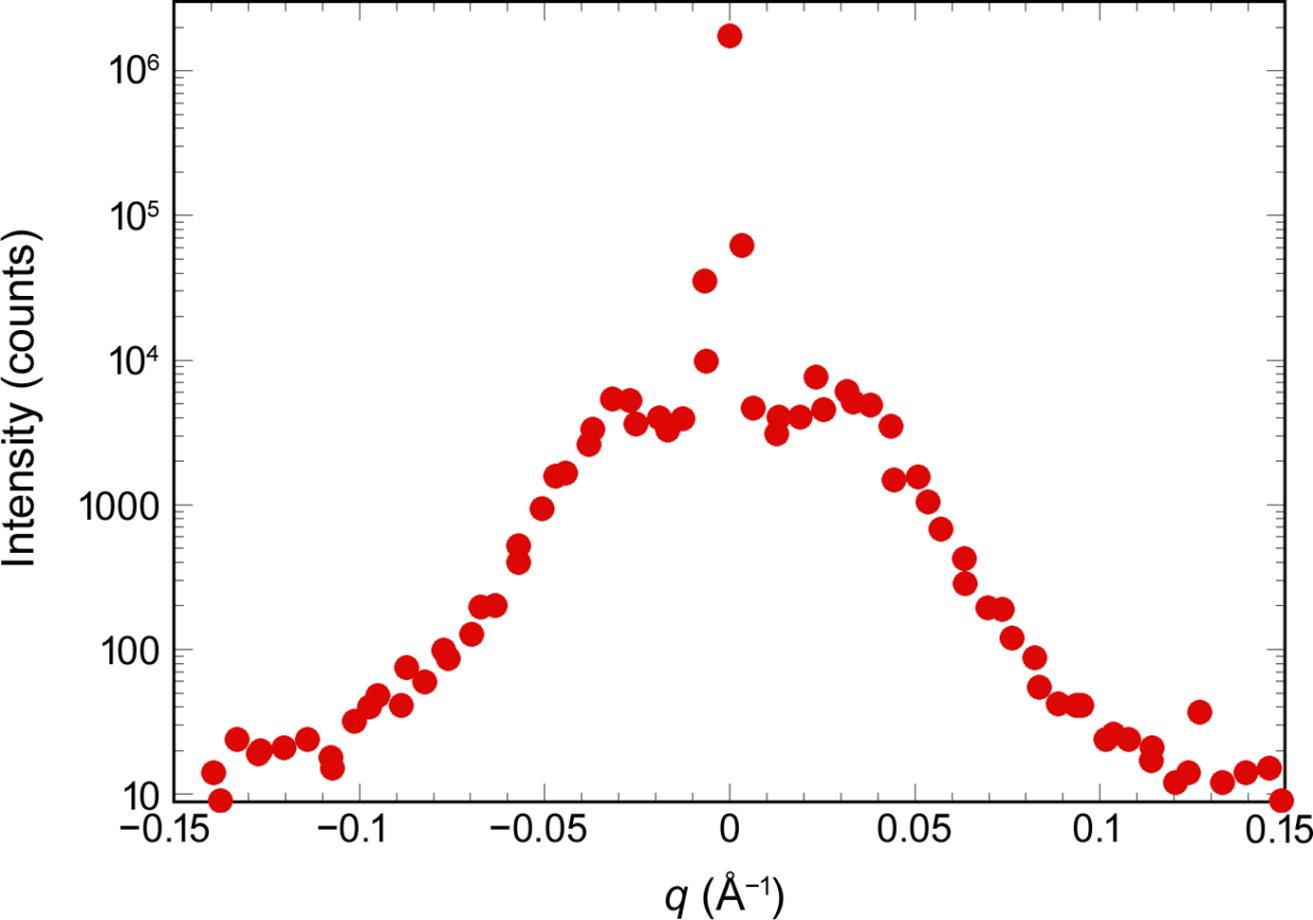
Reciprocal space slice taken along the fivefold axis around the 7/11 Bragg peaks (see Fig. 2 ▸). The intensity is displayed on a logarithmic scale. Two satellite reflections are clearly visible at about ±0.03 Å^−1^. The shape profile of the satellites is close to a Lorentzian and much broader than the main Bragg peak one.

By measuring the positions of the 12 satellites around the main Bragg peak accurately we found a modulation wavelength equal to 19.5 nm, *i.e.* very close to that observed in the synthetic AlCuFe quasicrystal (Menguy *et al.*, 1993 ▸*b*). The icosahedrite phase is thus a modulated phase, with a rather long modulation wavelength. We also observe that the satellites are much broader than the main reflections and are elongated along the fivefold axis, forming an ellipsoid-like shape similar to previous observations (Menguy *et al.*, 1993 ▸*b*). This is exemplified in Fig. 4 ▸, which displays a slice of the reciprocal space along the fivefold axis for the 7/11 fivefold axis reflection: satellites are visible on both sides of the main Bragg peak at about 0.3 nm^−1^. The satellites have a rather broad extension, close to a Lorentzian shape, whereas the main Bragg peak has a sharp Gaussian shape.

### 3.2. Analysis of the icosahedral modulated phase

Following the analysis of the modulated phase developed by Menguy *et al.* (1993 ▸*b*), we assumed that the modulation is the result of a cosine periodic distortion of the ideal icosahedral quasicrystalline phase by six waves propagating along the fivefold 10000 direction and its equivalent in 6D space, with the wavevector **q**_sat-6D_ = (**q**_par_, **q**_per_) and modulus *q*_par_ = *q*_per_ = 0.032 Å^−1^ corresponding to a very long wavelength of 19.5 nm. The strain wave has a polarization only in perpendicular space. Symmetry considerations impose the polarization to be along a fivefold direction in perpendicular space (Perez-Mato & Elcoro, 1994 ▸), which has been checked experimentally. The intensity distribution of the satellite intensity can then be calculated in a way similar to what is done for periodic modulated phases, *i.e.* using various orders of the Bessel function. For modulations with a relatively small amplitude the expression simplifies, and each first-order satellite reflection has a structure factor, *F*_sat_, given by
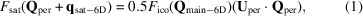
where **U**_per_ is the polarization of the phason modulation, **Q**_per_ is the perpendicular component of the associated main Bragg reflection and *F*_ico_(**Q**_main-6D_) is the structure factor of the main Bragg peak associated to the satellites and whose coordinate in reciprocal space is a node of the 6D reciprocal lattice given by **Q**_main-6D_ = (**Q**_par_, **Q**_per_).

**Figure 5:**
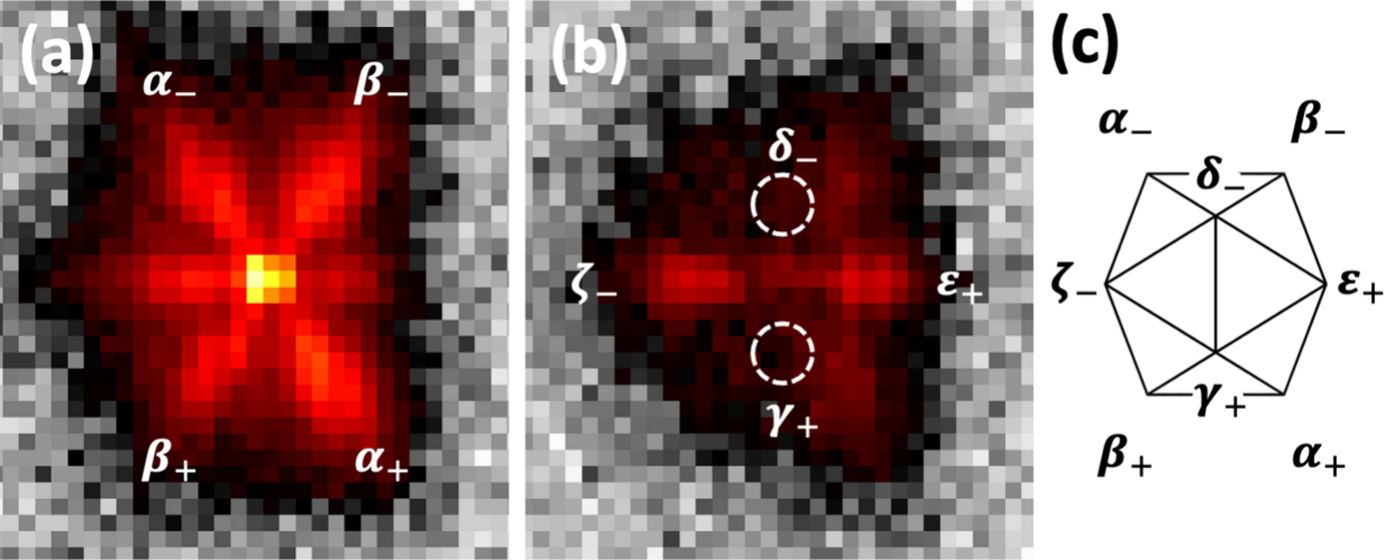
(*a*) Intensity distribution of the satellite reflections around the twofold 8/12 main Bragg reflection and (*b*) that at the slice just above Δ = 0.298 nm^−1^. (*c*) Layout of the satellite reflections. As expected, an extinction is observed for the directions δ and γ. Weak and strong satellites are observed for (ε, ζ) and (β, α), respectively. The + and − signs refer to the positive or negative positions in reciprocal space along the fivefold axis with respect to the main Bragg peak. Both panels (*a*) and (*b*) are of the same size: 1.66 nm^−1^ wide and 1.84 nm^−1^ high.

It can be easily realized that the satellite intensity distribution depends strongly on the scalar product **U**_per_ · **Q**_per_. A polarization **U**_per_ of the wave along a fivefold axis in perpendicular space implies that the satellites have their intensities grouped in three categories around a twofold reflection: extinct, weak and strong. A detailed representation of the intensity distributions of satellite reflections around a twofold, threefold and fivefold Bragg peak can be found in Menguy *et al.* (1993 ▸*b*). We have carefully checked that the intensity distribution indeed follows this scalar product constraint. An example is given in Fig. 5 ▸: as expected for a fivefold phason modulation around a twofold main reflection, satellite intensities are grouped in the three aforementioned categories. We have checked that the expected intensity distribution is indeed observed for Bragg peaks with different symmetries. We note that the achieved *Q*-resolution, unlike the experiment of Menguy *et al.* (1993 ▸*b*), was not sufficient for proper integration of the satellite intensities. Instead, an approximate evaluation of each satellite’s integrated intensity has been made by taking the maximum of the intensity distribution of each satellite reflection compared with the highest peak of the main Bragg peak, taking into account their width.

**Figure 6:**
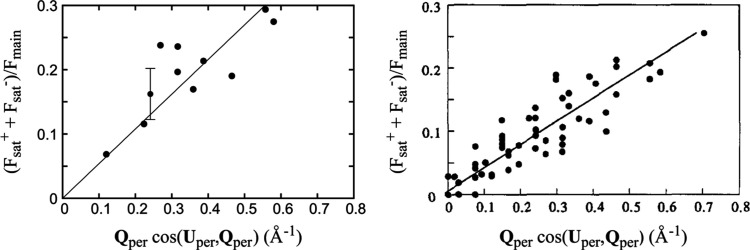
Evolution of the ratio of the satellite structure factors over the main Bragg peak ones as a function of the scalar product *Q*_per_ cos(**U**_per_, **Q**_per_) for the natural (left) and synthetic quasicrystals [right, extracted from Menguy *et al.* (1993 ▸*b*)]. In both cases there is a clear linear dependency. The slope is almost identical in both cases, indicating that the amplitudes of the modulation polarizations are similar.

From the measured data we have extracted a series of satellite intensities and their counterpart Bragg peak intensities in order to evaluate the modulus of the polarization **U**_per_. This is better achieved by plotting the ratio of the sum of pairs of satellite structure factors along each fivefold axis with the main Bragg peak structure factor as a function of the inner product **U**_per_ · **Q**_per_ in expression (1), which can be written as *Q*_per_cos(**U**_per_, **Q**_per_). The results are shown in Fig. 6 ▸ and compared with the results obtained in Menguy *et al.* (1993 ▸*b*). In both cases, a clear, almost linear, dependency is observed with a value of the *U*_per_ polarization of the same order of magnitude, *i.e.* 0.50 and 0.35 Å for icosahedrite and synthetic *i*-AlCuFe, respectively.

Furthermore, similar to that observed in Menguy *et al.* (1993 ▸*b*), an intensity difference is found between pairs of satellites located along the same fivefold axis. The maximum and minimum intensities alternate between positive and negative positions, depending directly on the sign of the **Q**_per_ component of the main Bragg peaks. Two tentative explanations for this behaviour have been proposed in Menguy *et al.* (1993 ▸*b*) but remain to be checked.

### 3.3. Icosahedrite crystallinity quality: mosaic spread and linear phason strain

It is interesting to compare the icosahedrite crystal quality to the samples obtained in the laboratory by slow cooling from the melt. Two parameters can be used: the mosaic spread of the single-crystal data and the residual phason strain that shows up as a residual Bragg peak broadening proportional to the *Q*_per_ component of the Bragg peak reciprocal 6D vector (Lubensky *et al.*, 1986 ▸).

**Figure 7:**
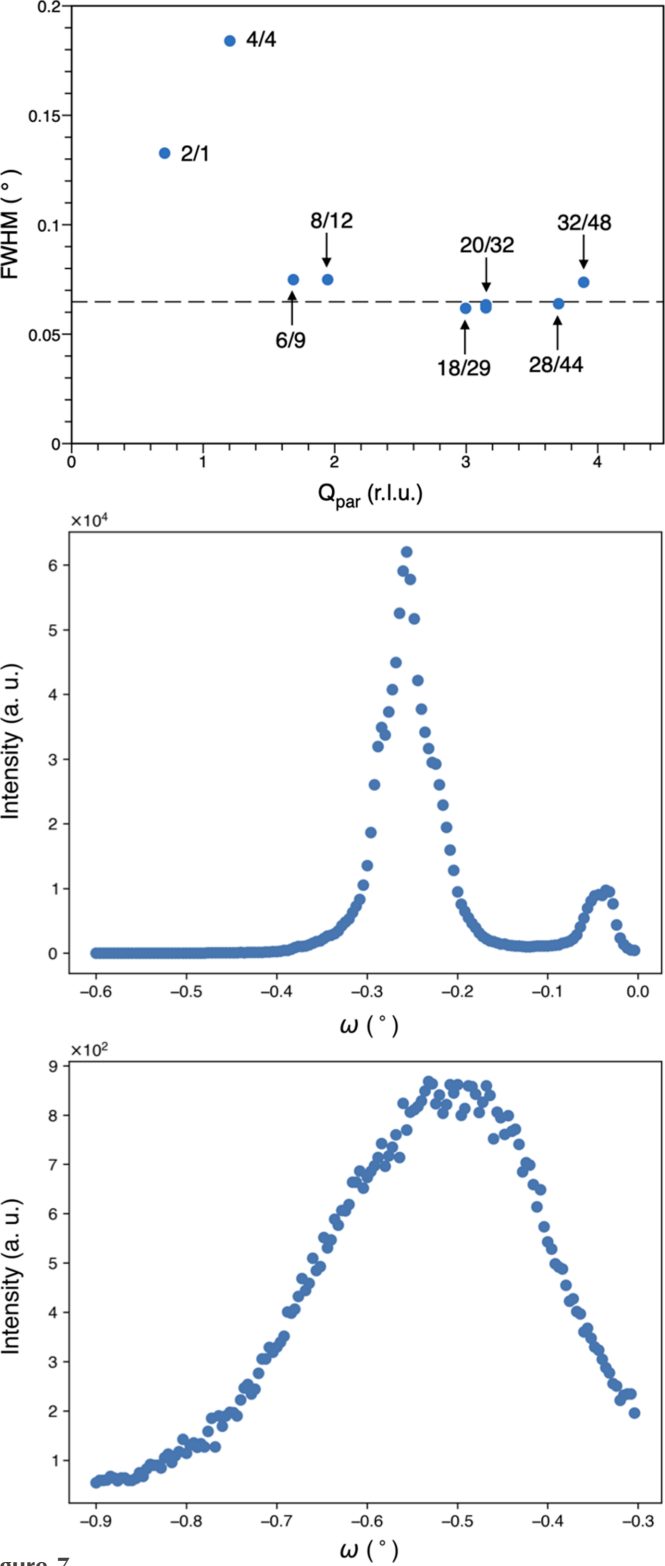
Upper panel: rocking curve width (FWHM) as a function of *Q*_par_ for a few Bragg peaks, indexed with their N/M indices. All widths are dominated by a mosaic spread equal to 0.065° except for two low-*Q* Bragg peaks. Central and bottom panels show the rocking curve for the 18/29 (*Q*_per_ = 0.18) and 4/4 (*Q*_per_ = 0.7) reflections with a clear broadening for the latter one.

Experimentally, the mosaic spread of single-grain quasicrystals strongly depends on the experimental systems considered and typical values span from 0.005° to 0.1° (Gastaldi *et al.*, 2003 ▸). For instance, the mosaic spread of the synthetic *i*-AlCuFe modulated phase was found to be equal to 0.02°. We have performed rocking curve scans around a few Bragg peaks, chosen to be close to the equatorial plane where the resolution is best and equal to the beam divergence in the vertical direction of 0.01°. After careful centring of each Bragg peak on the detector and selection of the appropriate attenuator, rocking curves have been measured by varying the omega angle of the diffractometer with 0.004° steps. By integrating the central part of the peak, typically 3 × 3 pixels at a detector distance of 225 mm, we found that the rocking curve width is equal to about 0.07°, which is larger than the instrumental resolution (0.01°) and similar to that observed for many quasicrystalline systems. An example of the rocking curve of the very strong fivefold 18/29 reflection is shown in Fig. 7 ▸. However, equivalent reflections under the icosahedral symmetry displayed a distribution of values of the mosaic spread from 0.07° to 0.5° depending on the sample orientation with respect to the beam.

When the instrumental resolution is good enough, a linear phason strain Bragg peak broadening has been observed for all quasicrystal phases (Gastaldi *et al.*, 2003 ▸). It depends on the growth conditions, sample annealing, system *etc*. Most likely this residual strain is due to a distribution of dislocations formed during the growth process, since dislocations in quasicrystals have both lattice and phason distortion fields associated (Levine *et al.*, 1985 ▸; Socolar *et al.*, 1986 ▸). The best results in this perspective have been obtained for the *i*-AlPdMn phase by comparing single grain samples obtained by the Czochralski method, in the as-grown state and after long time annealing. Although the annealing and very slow cooling down to room temperature did remove most of the dislocations, a very small residual phason strain was still present in the sample (Gastaldi *et al.*, 2003 ▸). The evaluation of an eventual phason strain broadening of the Bragg peaks thus requires a very good *Q*-resolution (Gastaldi *et al.*, 2003 ▸). The instrumental *Q*-resolution depends on the beam divergence, beam energy, energy resolution of the monochromator, sample-to-detector distance and pixel size in the case of a 2D detector. With the current settings, the beam divergence is 0.01° and Δ*E*/*E* is better than 10^−4^. Close to the equatorial plane, and for low 2θ diffraction angles, the transverse *X* and *Y* resolution on the detector is dominated by the pixel size and is equal to about
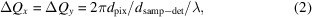
where *d*_pix_ is the pixel size equal to 0.18 mm, *d*_sample–det_ is the sample-to-detector distance equal to 244 or 414 mm, and λ is the X-ray wavelength. The *Q*-resolution on the detector is thus of the order of 0.008 and 0.005 Å^−1^ for the short and long detector distances, respectively (as for the rest of the paper, we have chosen the convention *Q* = 4πsin(θ)/λ to calculate all reciprocal space values).

In principle, a much better resolution is achieved when carrying out an omega scan. The instrumental resolution is then given by the beam divergence and can be written as
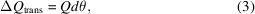
where *d*θ is the beam divergence expressed in radians. It can be readily realized that the best resolution is achieved for low-*Q* reflections. For instance, for a reflection at *Q* = 1 Å^−1^, the instrumental resolution is equal to 0.0002 Å^−1^, *i.e.* 40 times better than on the *X* and *Y* detector directions. To this contribution, we need to add the sample mosaic spread contribution, which dominates the resolution in this configuration. Assuming a mosaic spread of 0.065°, one obtains a transverse resolution equal to 0.001 Å^−1^ at 1 Å^−1^ and 0.003 Å^−1^ at 3 Å^−1^.

**Figure 8:**
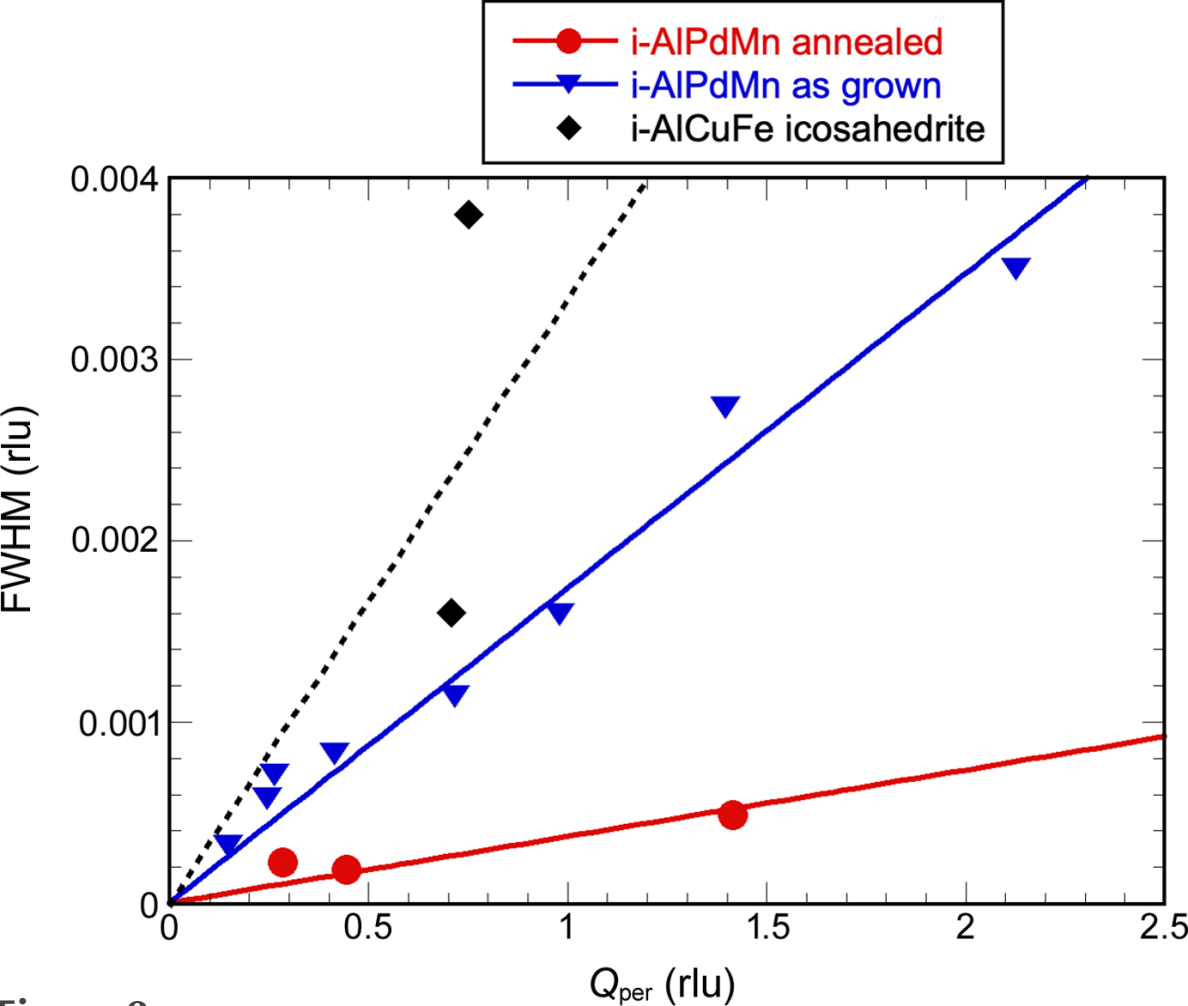
Comparison of the Bragg peak width (FWHM) as a function of *Q*_per_ for the *i*-AlPdMn phase in the as-grown (blue triangles) and annealed [red circles, from Gastaldi *et al.* (2003 ▸)] states, together with an estimate for the icosahedrite (black diamonds). Both the width and *Q*_per_ are expressed in 2π/*a* units (rlu).

We have measured omega scans of a series of Bragg peaks having a *Q*_per_ value in the range 0.17 to 0.7 Å^−1^. Each Bragg peak has been centred prior to the omega-scan measurement. As shown in Fig. 7 ▸, most Bragg peaks have a width given by the mosaic spread equal to 0.065°. Only two low-angle Bragg peaks depart significantly from this value. This is not enough to give an accurate estimation of the linear phason strain, but an estimate can be extracted and compared with other values. The phason strain broadening is extremely small, and just above the value obtained in the as-grown sample of the *i*-AlPdMn phase (Gastaldi *et al.*, 2003 ▸), as shown in Fig. 8 ▸. The as-grown *i*-AlPdMn was obtained by the Czokralski method with a very slow pulling rate (Boudard *et al.*, 1995 ▸), it is thus quite remarkable that the quality of the natural quasicrystal is so good both in terms of mosaic spread and residual linear phason strain.

## 4. Discussion

It is quite extraordinary that the single grain of icosahedrite studied here is nearly identical to the synthetic AlCuFe icosahedral modulated phase observed as a pre-transitional state towards the microcrystalline state. The fact that the wavelength and the amplitude of the modulation, polarized in the phason space, are the same in both samples indicates that the studied icosahedrite fragment is homogenously modulated. This is also confirmed by the sample height *z*-scans that were performed, and for which the same modulated diffraction pattern has been observed for all the *z* positions. From the analysis of other high-pressure phases present in the meteorite, we know that icosahedrite likely formed in the pressure range from 5 to 10 GPa.

It has been shown that pressure influences the stability of the icosahedral phase in the Al–Cu–Fe system both under static (Stagno *et al.*, 2014 ▸, 2015 ▸, 2017 ▸, 2021 ▸; Stagno & Bindi, 2023 ▸) and dynamic conditions (Asimow *et al.*, 2016 ▸; Oppenheim *et al.*, 2017*a* ▸,*b* ▸; Hu *et al.*, 2020 ▸). It was demonstrated that at 5 GPa the melting point of icosahedrite is about 500 K higher than at ambient pressure and shock-gun experiments produced icosahedral AlCuFe quasicrystals with compositions outside the reported stability field, indicating a change in the phase boundaries of Al–Cu–Fe *i*-phases under impact conditions. Given these circumstances, it is very hard to make general considerations on the recovered modulated phase. However, we can attempt to speculate, taking into account a not so different behaviour from the known one at ambient pressure. The growth of the quasicrystal from the melt is driven by the peritectic reaction leading to single grains with a very narrow chemical composition range. For this specific chemical composition and from previous *in situ* and *ex situ* studies it is also known that the formation of the low-temperature phase is due to a phason softening leading to a fivefold instability. From the high-temperature icosahedral phase, the two phason elastic constants characterizing the icosahedral quasicrystal soften with a positive ratio leading to a fivefold instability and the icosahedral modulated phase (Widom, 1991 ▸; Ishii, 1989 ▸, 1990 ▸, 1992 ▸; Menguy *et al.*, 1993 ▸*b*). A further slow cooling leads to the rhombohedral periodic phase which forms around 600°C (Audier & Guyot, 1989 ▸, 1990 ▸). This phase transition is reversible as shown by *in situ* studies (Bancel, 1989 ▸, 1993 ▸; Boudard *et al.*, 2000 ▸), confirming the positive ratio between the two phason elastic constants (Boudard *et al.*, 2000 ▸). Moreover, the kinetics of formation of the modulated phase around 600°C are relatively slow, and its quenching to room temperature is relatively easy, as shown experimentally by Audier *et al.* (1991 ▸). The formation of the modulated phase could thus in principle be used as a ‘temperature tracer’ to infer a possible temperature profile of the formation of the studied natural quasicrystal single grain. One possible scenario (inferred from ambient-pressure experiments) is that, under the shock and at high temperature, the quasicrystal formed and grew as a single crystal in the range 850–800°C, followed by relatively slow cooling to about 600°C, at which point cooling to room temperature was likely to be faster.

Finally the present results can be compared with the electron diffraction patterns collected on an icosahedrite fragment coming from another part of the same meteorite (Bindi *et al.*, 2009 ▸). No trace of the modulation was observed in this diffraction pattern. This might be related to the lower resolution of the setup that makes the detection of the very small modulation wavevector difficult. Alternatively, it could also result from the large inhomogeneities observed throughout the meteorite samples in term of pressure, temperature and thermal history. Evidence of this inhomogeneity includes the coexistence of both the high- and low-pressure phases of Mg_2_SiO_4_ (ringwoodite/olivine), and the presence of the high-pressure polymorph of SiO_2_, *i.e.* stishovite in the Khatyrka meteorite (Hollister *et al.*, 2014 ▸)

It is thus likely that both the icosahedral high-temperature phase and the modulated icosahedral phase coexist in the Khatyrka meteorite.

## 5. Conclusions

Using high-resolution synchrotron X-ray diffraction, we have studied a single grain of icosahedrite, a natural *i*-AlCuFe quasicrystal. The investigated fragment is in a modulated icosahedral quasicrystal state, similar to that observed previously in a synthetic icosahedral quasicrystal with the same composition. The modulated phase is described by the distortion of the ideal icosahedral quasicrystal by six cosine phason waves propagating along the six fivefold symmetry axes and having a polarization uniquely in the perpendicular phason space. The wavelength of the modulation, equal to 19.5 nm, and its amplitude along the fivefold axis in perpendicular space of the order 0.05 nm are similar in both natural and synthetic quasicrystals. The single grain of icosahedrite examined in this study is in a pre-transitional state due to a fivefold phason softening. It has most likely been formed by slow cooling from the liquid state followed by quenching to lower temperature. Despite all the limitations, the observation of the modulated state in icosahedrite could thus in principle be used as a tracer of the thermal history of the meteorite. The diffraction quality is also very good with a narrow mosaic spread and a rather weak residual linear phason strain, as observed by the Bragg peak broadening. However, the pressure and temperature are inhomogeneous in the meteorite as exemplified by the presence of both high- and low-pressure polymorphs of Mg_2_SiO_4_. This implies that both the icosahedral quasicrystal and the modulated phase could plausibly coexist in Khatyrka meteorite remnants.

## Data Availability

After the embargo period, all raw data will be available at https://doi.org/10.15151/ESRF-ES-1108359197. Analysed data can be obtained upon request.
